# Chains of Nanoparticles for Flat-Band Emission and Lasing

**DOI:** 10.1021/acs.nanolett.6c01138

**Published:** 2026-04-23

**Authors:** Rebecca Heilmann, Joel Lehikoinen, Sioneh Eyvazi, Evgeny A. Mamonov, Päivi Törmä

**Affiliations:** † Department of Applied Physics, Aalto University School of Science, P.O. Box 15100, Aalto FI-00076, Finland

**Keywords:** flat bands, plasmonics, nanoparticle arrays, lasing

## Abstract

Controlling light–matter interactions is central to photonic technologies ranging from lasers to optical information processing. Suitably designed photonic structures give rise to flat bands, where the density of states diverges and the group velocity goes to zero, allowing light localization. These properties make flat bands attractive for lasing; however, designing photonic structures that support flat bands is challenging. Here, we introduce long-range coupled chains of nanoparticles that support totally flat bands extending over the full angular range. We demonstrate flat-band lasing in single chains and explain the transition to Γ-point lasing as the number of chains is increased. Moreover, we show partially coherent emission from square and triangular two-dimensional chain lattices. The excited modes depend on the pump power and polarization. Our results establish chain lattices as a versatile platform for exploring flat-band lasing and suggest new routes toward narrow-band, linearly polarized, and bright light sources with tailored coherence.

## Graphical abstract

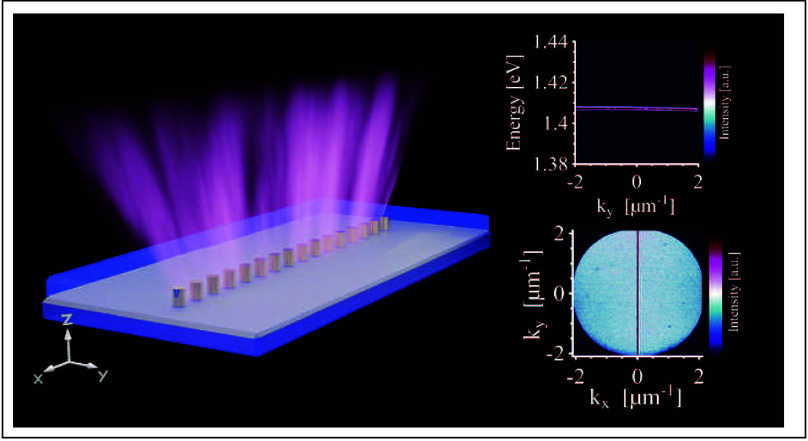

Periodic photonic structures provide a means to tailor light–matter interactions by engineering the underlying band structure. While most photonic bands are dispersive, suitably designed lattices can host flat bands, i.e., bands of dispersionless modes whose energy remains constant over a range of momenta. Photonic and exciton–polariton systems with flat bands have attracted interest^1–3^ because they enable experimental realizations of Hamiltonians associated with exotic topological many-body phenomena, including the fractional quantum Hall effect,^4^ superconductivity,^5^ and ferromagnetism.^6^ Their nondispersive nature also gives rise to a diverging density of states, vanishing group velocity, and compact localized eigenstates arising from destructive interference,^1^ which in turn strengthen nonlinear optical processes,^7,8^ give rise to slow light effects,^9^ and offer control over and enhancement of emission^10^ and absorption.^11^ The properties described above make flat-band platforms particularly attractive for coherent light generation. Localized states act as intrinsic optical cavities; the low group velocity increases the photon lifetime and effective quality factor (*Q*-factor), and the high density of states increases emission rates, reducing the lasing threshold. However, designing flat-band photonic systems is challenging, as long-range couplings, typical in photonic systems, tend to induce dispersion. Coherent emission from a flat band was accordingly first observed in exciton–polariton systems with Lieb or kagome tight-binding (nearest-neighbor-coupled) lattices.^12–17^ For photonic systems, a theoretical description of a flat-band laser in an evanescently coupled (tight-binding) system was proposed by Longhi.^18^ By combining tight-binding and long-range effects, multiple scattering theory has been used to predict that one-dimensional (1D) chains of high-index spheres with a specific chain spacing give rise to a flat band.^19^ Another noticeable system showing lasing is a moiré lattice realized by two twisted hexagonal geometries,^20^ where twist-induced destructive interference creates a high-*Q* flat-band nanocavity. This platform was subsequently extended to reconfigurable nanolaser arrays with programmable emission patterns.^21^ Other approaches have sought to leverage the inherently high *Q*-factors of quasi-bound states in the continuum (for the sake of brevity, BICs) in conjunction with flat bands. Eyvazi et al. employed a silicon metasurface to couple out guided modes, whose dispersion is flattened by the contrast between the effective refractive index of the guided mode and its surroundings.^22^ They also observed symmetry-protected and accidental BICs on the flat band. Do et al. carefully tuned the interaction of four guided modes via the anisotropy of a rectangular metasurface to locally flatten a band with a BIC at the Γ-point, leading to a flat mode with a *Q*-factor of more than 9000 in the visible range.^23^ Cui et al. designed a lattice featuring a symmetry-protected BIC at the Γ-point surrounded by accidental BICs yielding a flat band in terahertz spectral region.^24^

In the companion manuscript,^25^ we show by theory that 1D chains of nanoparticles and two-dimensional (2D) lattices constructed from them, termed chain lattices, host flat bands. Contrary to the previous photonic flat-band realizations,^20–24^ these flat bands arise purely from diffraction in a non-tight-binding (long-range-coupled) system. Furthermore, they extend over all momenta. Here, we investigate the optical properties of selected chain lattice geometries. We present how chain lattices transition from a flat-band hosting structure to a regular 2D square lattice with TE- and TM-polarized surface lattice resonances (SLRs) as the array size changes in terms of dispersion and lasing. We study emission from square and triangular 2D chain lattices and show how the spatial- and momentum-space emission patterns depend on the array geometry, pump intensity, and its polarization. We find coherence within individual chains, while the 2D lattice as a whole produces bright, incoherent radiation. Our findings establish single chains and their lattices as a versatile platform for realizing both coherent and incoherent emission from flat bands.

We realized the lattices under study by using metallic plasmonic nanoparticles embedded in a gain material. Plasmonic nanoparticle arrays^26,27^ host collective modes called surface lattice resonances (SLRs), which combine diffractive orders and single-nanoparticle resonances, and are well suited for realizing the essential features of the chain lattice flat bands proposed in ref 25. Moreover, the SLRs can provide optical feedback for the gain medium, resulting in lasing.^28–31^ The extinction and emission spectra of the arrays are studied using momentum-resolved spectroscopy, and the spatial coherence of the emission is investigated via Michelson interferometry.

We first consider the band structures of arrays that are effectively infinite in the *x*-direction with periodicity *p* and have *L* chains (rows) in the *y*-direction with the same distance *p* between them (see Figure 1a). Intuitively, for a single chain, we expect its dispersion *E*(*k*_*y*_) at *k*_*x*_ = 0 to be flat, because the periodic structure in the *x*-direction permits constructive interference only at specific energies and wavevectors **k**_*x*_, whereas the lack of structure in the *y*-direction forces the band to be flat in that direction. On the other hand, in the limit of a large *L*, we expect the dispersion to approach that of a square lattice, which does not have any flat bands; instead, it shows dispersive TE- and TM-polarized SLR modes. This raises the question of the values of *L* at which the band structure transitions from the flat dispersion of a single chain to that of the square lattice. We answer this question by studying arrays of gold nanoparticles using momentum-resolved transmission spectroscopy and by calculating the band structures of the lattices using the empty lattice approximation (for more information on sample fabrication, characterization, and theory, see the Supporting Information).

**Figure 1:**
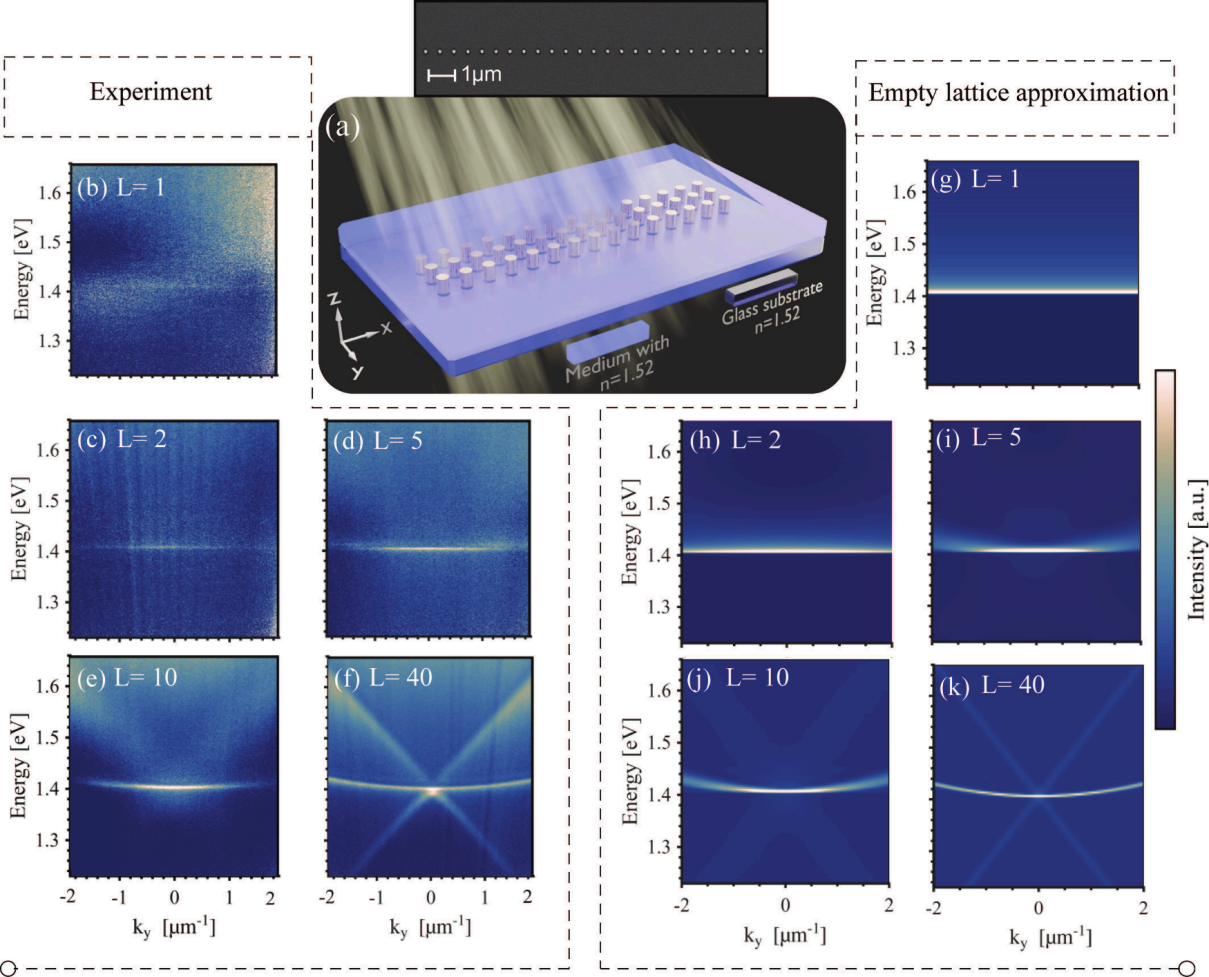
Experimental measurement of the extinction. (a) Scheme of a number (*L*) of parallel chains. The *L* = 3 case is depicted in the schematic, while the inset shows a scanning electron microscope image of a single chain (*L* = 1). (b–f) Measured extinctions and (g–k) band structures calculated using the empty lattice approximation of *L* = 1, 2, 5, 10, and 40 lines, respectively, of square lattice with a periodicity of 580 nm. Particles were gold nanocylinders with a diameter of 120 nm and a height of 50 nm in an index-symmetric background with *n* = 1.52, and no polarization filters were used in the measurement. The first Brillouin zone (for an *L* → *∞* system) extends between *k*_*y*_ values of approximately −5.4 and 5.4 μm.

Figure 1 shows the experimentally obtained dispersions (panels b–f) and band structures calculated using the empty lattice approximation (panels g–k) of lattices with *L* = 1, 2, 5, 10, and 40 chains, respectively. The chains consist of cylindrical gold nanoparticles with a diameter of 120 nm and a height of 50 nm with a chain periodicity of 580 nm. The periodicity was chosen such that the energy of the flat band overlaps with the gain medium in the lasing experiments such that lasing can be achieved. The size and the shape of the nanoparticles were selected to optimize the excitation of the plasmon resonances. The experimental data are in excellent agreement with the theory. The expected transition from a flat band for a small *L* to the curved TM mode of a square lattice at a larger *L* is clearly visible, and the cross-like TE modes of a square array become visible when *L* = 40.

The flat band in the single-chain case is intuitively understood as the *x*-direction discrete translational invariance (lattice periodicity) fixing the wavelength of the light, while the *y*-direction momentum is not limited since the narrow chain breaks translational invariance in the *y*-direction. In the 2D arrays, there is periodicity also perpendicular to the chains but with a large unit cell. The TM modes in the replicas of the corresponding small Brillouin zones form the flat bands. It can also be formally explained by the light dispersion in a 2D rectangular periodic system

1 $$E\left(k_{∥}\right)=\frac{\hbar c_{0}}{n}\sqrt{{\left(k_{x}+m\frac{2\pi }{a_{x}}\right)}^{2}+{\left(k_{y}+m^{\prime }\frac{2\pi }{a_{y}}\right)}^{2}}$$

where $k_{∥}={\left(k_{x},k_{y}\right)}^{T}$ , *ℏ* is the reduced Planck’s constant, *c*_0_ is the speed of light in a vacuum, *n* is the refractive index, *a*_*x*_ and *a*_*y*_ are periods, and *m* and *m*′ are integers. Let us consider the case in which *E* ∼ 2*πℏc*_0_/(*na*_*x*_), i.e., diffraction (*m* = ±1) in the *x* direction, and *k*_*x*_ = 0. The single chain corresponds to *a*_*y*_ → ∞; there are thus infinitely densely spaced values of *m*′2π/*a*_*y*_ to match any values of *k*_*y*_ so that the energy remains the same, producing a flat band. For a finite *a*_*y*_, the first diffracted order *m*′ = ±1 would correspond to a quite different energy, but *m*′ = 0 gives approximately parabolic dispersion $E=\left(\hbar c_{0}/n\right)\sqrt{{\left(2\pi /a_{x}\right)}^{2}+k_{y}^{2}}$ close to the energy *E* ∼ 2*πℏc*_0_/(*na*_*x*_). In a transition between a single chain and a full 2D lattice, both the flat band and the parabolic TM mode are visible in the experiments and theory of Figure 1; both of these are determined by the first diffracted order in the *x* direction. The cross-type TE mode that appears for *L* = 40 corresponds to the first diffracted order in the *y*-direction (*m*′ = ±1; *m* = 0), giving a linear dispersion *E* = (*ℏc*_0_/*n*)­(*k*_*y*_ ± 2π/*a*_*y*_) (in our example, *a*_*x*_ and *a*_*y*_ are set to the same value).

We study the lasing emission from the flat-band mode by combining the nanoparticle chains with an organic gain medium (IR 140, 10 mM) and pumping this system optically with a 100 fs pulsed laser at a central wavelength of 800 nm (see the Supporting Information for details of sample fabrication and measurements). We first studied the single-chain system in Figure 2 to understand lasing within a single building block of the system. The emission spectrum above the threshold is shown in Figure 2b, where the emission clearly stems from the flat-band mode observed in Figure 1b. With an increase in pump fluence, we observe an exponential increase in intensity and a prominent narrowing of the line width at threshold (see Figure 2c), providing a *Q*-factor of 900. The real- and momentum-space emission are shown in panels d and e, respectively, of Figure 2. From Figure 2e, it is evident that the emission covers the whole range of *k*_*y*_ while at the same time being confined tightly around *k*_*x*_ = 0. We measured the spatial coherence of the single-chain emission via Michelson interferometry; for a description of the method, see the Supporting Information. The emission is coherent as there are clearly visible fringes in the interference pattern (Figure 2h), and hence, the system is lasing. We also note that Rekola et al. studied single chains of nanoparticles;^32^ however, they focused on the dispersion along the chain (here *k*_*x*_), which does not show a flat band.

**Figure 2:**
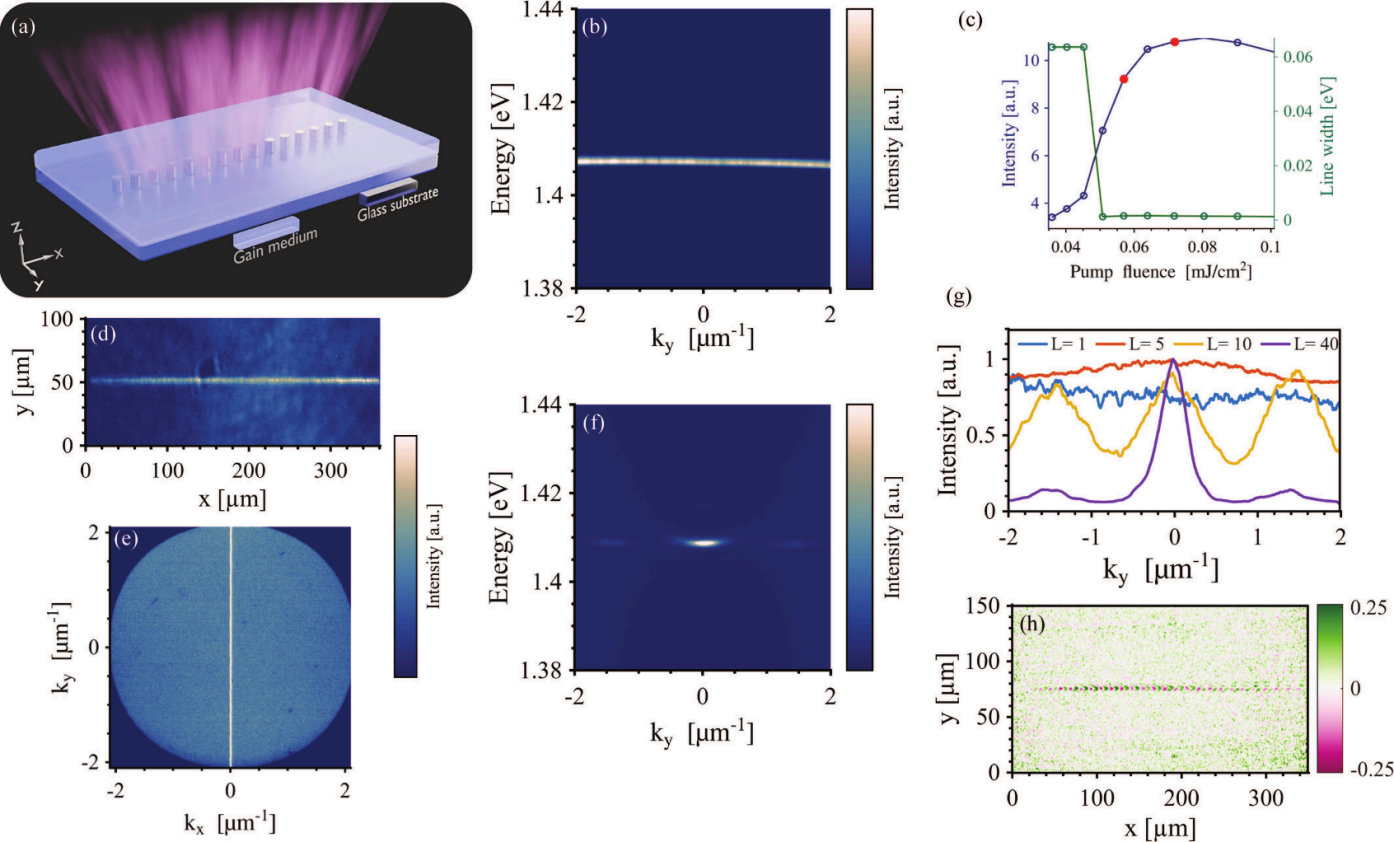
Lasing emission from single-chain systems. (a) Scheme of the single chain. (b) Momentum-space-resolved spectrum of single-chain system lasing emission at a pump fluence of 0.05 mJ/cm^2^. (c) Dependencies of the emission intensity and line width on pump fluence for a single-chain system at a pump fluence of 0.07 mJ/cm^2^. (d and e) Real- and momentum-space emission patterns, respectively, of single-chain lasing. (f) Momentum-space-resolved spectrum of *L* = 40 chain system lasing emission at a pump fluence of 0.09 mJ/cm^2^. (g) Dependence of lasing emission on *k*_*y*_ for systems with different numbers of chains. The pump fluences were as follows: 0.05 mJ/cm^2^ for *L* = 1, 0.07 mJ/cm^2^ for *L* = 5, 0.1 mJ/cm^2^ for *L* = 10, and 0.09 mJ/cm^2^ for *L* = 40 (h) Background-free Michelson interference pattern of single-chain lasing emission at a pump fluence of 0.1 mJ/cm^2^.

In addition to the single chains, we also studied ensembles of chains with different values of *L*. The emission spectrum of a lattice with *L* = 40 is shown in Figure 2f, where most of the emission is now at the Γ point with two side maxima. We observe that the lasing emission along *k*_*y*_ depends strongly on the number of chains (*L*), where the flat-band emission breaks down at *L* = 10 (Figure 2g). The side maxima visible for *L* = 10 and *L* = 40 are related to the system size in the *y*-direction.

Next, we study combinations of several chains in different configurations to tailor the direction of the flat band and to utilize the gain medium more efficiently than a single chain would. In our first example, chains that are combined to form a square 2D chain array (Figure 3a) show under optical pumping a nonlinear increase in emission intensity (Figure 3b) with a *Q*-factor of 200. The emission above the threshold stems from the flat-band TM mode similar to the single chains, as is evident from the momentum-resolved spectrum and the momentum-space emission pattern in panels c and e, respectively, of Figure 3. The flat-band mode is formed by the superposition of the SLR modes associated with the diffracted orders (±1, *m*′), $m^{\prime }\in Z$ . The real-space emission pattern shows that the emission originates mainly from the chains in the *x*-direction (see Figure 3d). Although the structure is *x*–*y*-symmetric, one direction is favored due to pump polarization (along the *y*-direction). For more information on the influence of pump polarization, see Figure S4. The interference pattern of the square 2D chain array (Figure 3f) shows clear coherence for the line in the center only. This means that we have coherent emission in part of the system; namely, each single line is coherent with itself and hence lasing. However, the system as a whole is not coherent, and the emission of different chains is not phase-locked.

**Figure 3:**
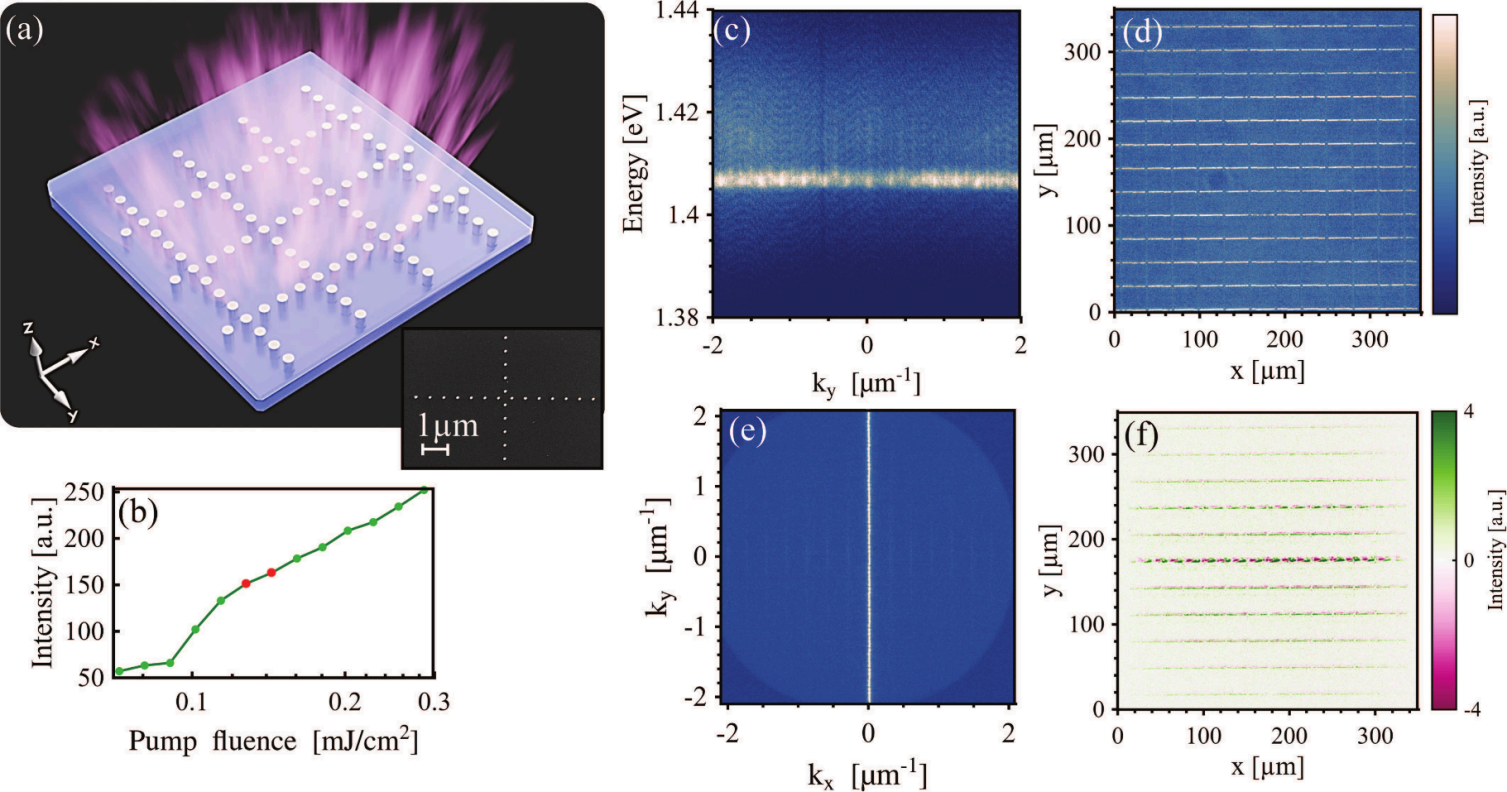
Emission from square 2D chain arrays. (a) SEM image and scheme of the square 2D chain array. (b) Dependence of emission intensity on pump fluence. (c) Momentum-space-resolved spectrum of square 2D array emission at a pump fluence of 0.13 mJ/cm^2^. (d and e) Real- and momentum-space patterns, respectively, of square 2D chain array emission at a pump fluence of 0.14 mJ/cm^2^. (f) Background-free interference pattern of the square 2D chain array emission, showing fringes along the *x*-direction but fading features in the *y*-direction. The pump fluence was 0.25 mJ/cm^2^. The arrays consisted of cylindrical gold nanoparticles with a diameter of 110 nm and a height of 50 nm. The period of the array was 580 nm, and there were 40 particles between the chains.

To demonstrate the versatility of the concept, we show in Figure 4 the emission of triangular 2D chain arrays, where chains that are oriented along three different angles combine to form an array (see Figure 4a for an SEM image and the scheme of the array). While the momentum-space-resolved spectrum again shows emission from the flat-band TM mode (Figure 4b), we clearly observe two thresholds in the emission intensity with an increase in pump fluence (Figure 4c). The real- and momentum-space emission patterns are shown for these two regimes in panels d and e, respectively, of Figure 4 for the lower pump fluence and panels f and g, respectively, for the higher pump fluence, At the lower pump fluence, only the chains along the *x*-axis are excited, showing emission only at *k*_*x*_ = 0 and for all *k*_*y*_ values (again, this direction is chosen by the *y*-polarized pump), whereas at the higher pump fluence, the diagonal chains are also excited, leading to flat-band emission also along angles other than *k*_*x*_ = 0, that is, *k*_*y*_ = ±tan(30°)*k*_*x*_ (these two regimes can also be observed for square 2D chain arrays (see Figures S4 and S5)). Again, the central lines are coherent with themselves, meaning that single chains are lasing, whereas the array as a whole shows incoherent emission. This is evident from the interference image shown in Figure 4h, which has fringes along only positions that correspond to coherence along the chains in three different directions.

**Figure 4:**
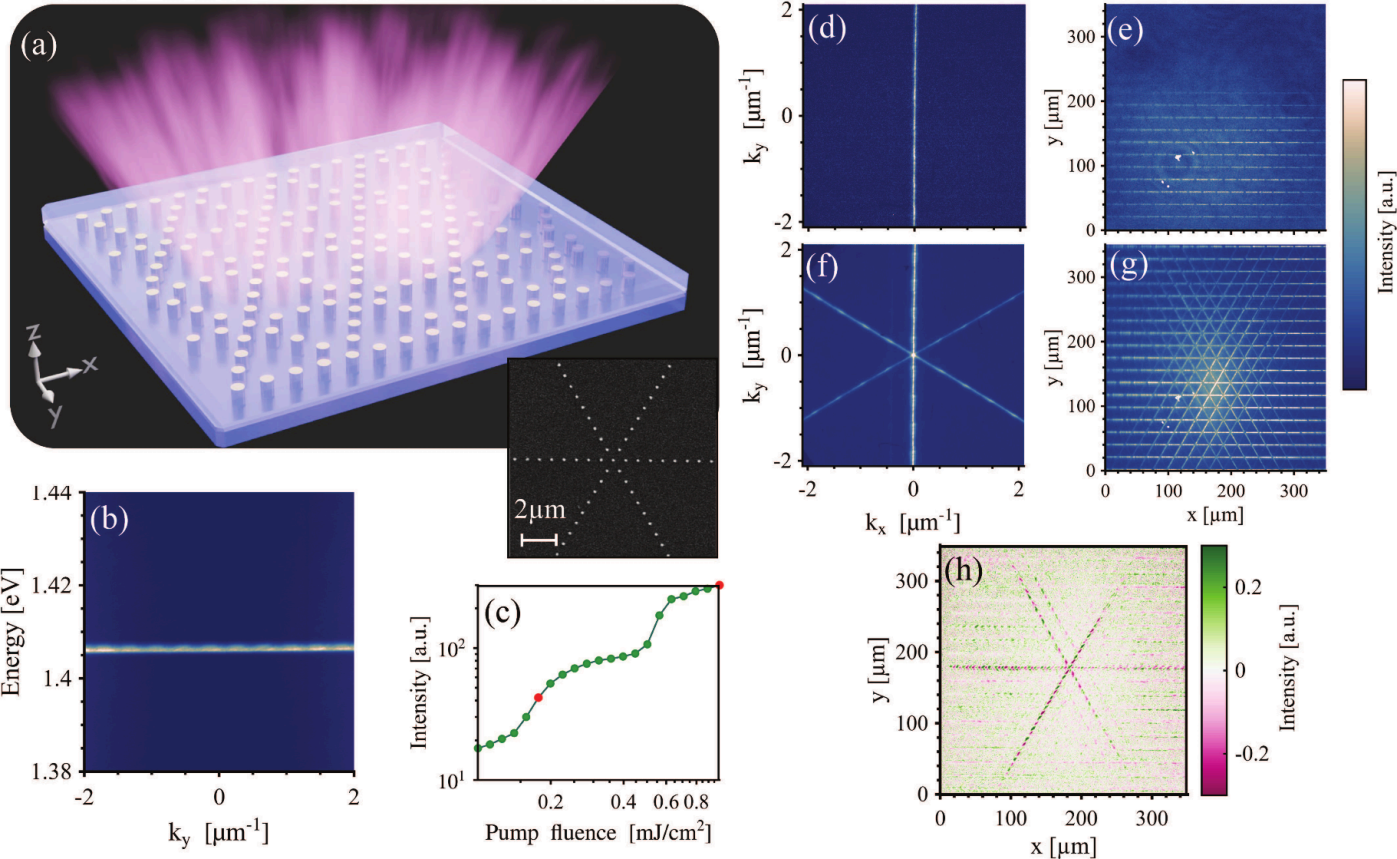
Emission from triangular 2D chain arrays. (a) SEM image and scheme of the triangular 2D chain array. (b) Momentum-resolved spectrum of the triangular array emission at a pump fluence of 0.18 mJ/cm^2^. (c) Dependence of emission intensity on pump fluence. The red dots denote pump fluence values used for momentum- and real-space pattern collection. (d and f) Momentum-space and (e and g) real-space emission patterns of triangular 2D chain arrays for fluence values of (d and e) 0.18 mJ/cm^2^ and (f and g) 1 mJ/cm^2^. (h) Background-free interference pattern of triangular 2D chain array emission under a fluence value of 1 mJ/cm^2^. The arrays consisted of cylindrical gold nanoparticles with a diameter of 120 nm and a height of 50 nm. The period of the chains was 580 nm, and the distance between the chains was 33 particles.

In summary, we have experimentally demonstrated that arrays built from single-nanoparticle chains are flexible platforms for bright, polarized light generation. The design of the chain arrangement allows realization of different axially symmetric flat-band patterns, and applying specific pump energies leads to effective excitation of only certain modes.

We first demonstrated the formation of a flat band in the TM mode of single chains of nanoparticles and experimentally observed lasing in this flat band. We showed that the emission of the single chains is also coherent when combining ensembles of chains into arrays. While the single building blocks are coherent, these parts are not mutually coherent. As such, this is useful as a bright, incoherent light source, while optimization of the *Q*-factors and chain couplings could be done to make the total emission coherent if desired. In addition, we showed that we can control the angles at which the flat band appears by arranging the chains accordingly. With a change in the pump fluence and pump polarization, specific modes of the system can be excited.

Because the flat bands in the chain lattices arise purely from diffraction,^25^ their appearance is not tied to a specific experimental platform. Due to the small amount of particles in the chains and 2D chain systems, sufficiently large particles are needed to obtain modes that are strong enough to experimentally realize flat bands. However, ohmic losses in plasmonic particles prevent lasing in chain systems of particles with diameters larger than 130 nm. Conceivably, due to the absence of ohmic losses, higher *Q*-factors could be reached for the flat-band modes using dielectric nanoparticles instead of metallic ones; the former also offer an additional design degree of freedom due to their magnetic resonances. Two key properties differentiate the chain lattices from the previously reported flat-band lasing realizations.^12–17,20–24^ First, other realizations of flat-band lasing systems with long-range coupling have relied on using guided modes,^22,23^ leading to either quasi-flat-band systems or flat bands within a finite angular extent. The flat bands reported here extend naturally to all angles. Second, here the appearance of flat bands does not require fine-tuning of the lattice parameters (e.g., to realize nearest-neighbor coupling); instead, the lattice parameters determine the spectral positions of the flat bands predictably and straightforwardly. Our experiments and the theory work of ref 25 thus introduce a promising complementary concept for further studies and applications of photonic flat bands.

## Supplementary Material



## Data Availability

The experimental raw data are available at 10.5281/zenodo.18314743.

## Notes

Sample fabrication, lasing and transmission measurement setup, spatial coherence measurement principle, theory, influence of pump polarization, *k*-space emission data from square chain lattices, and emission from 2D chain arrays with the IR792 dye (PDF)
