# Supplementary material for: Chains of Nanoparticles for Flat-Band Emission and Lasing
Source: Nano Lett. 2026 Apr 23;26(17):5960–6. doi: 10.1021/acs.nanolett.6c01138 (PMC13154355; doi:10.1021/acs.nanolett.6c01138)
Supplement: Supplementary file 1 [file nl6c01138_si_001.pdf]

Chains of nanoparticles for flat-band emission and lasing — Supplementary Material

Rebecca Heilmann[1], Joel Lehikoinen[1], Sioneh Eyvazi[1], Evgeny A. Mamonov[1], Päivi Törmä[1*]

[1]Department of Applied Physics, Aalto University School of Science, P.O. Box 15100, Aalto FI-00076, Finland.

*Corresponding author(s). E-mail(s): paivi.torma@aalto.fi;

Sample fabrication

The nanoparticle arrays were fabricated with electron beam lithography on borosilicate substrates ($n = 1.52$). A 2 nm layer of titanium was used for better adhesion of the gold nanoparticles to the substrates. The height of the nanoparticles was 50 nm, the particle diameter, period, and length of the single chains were 120 nm, 580 nm, and 300 µm. The particle diameter, period, and size of the 2D chain arrays were 110 nm, 580 nm, and 300 µm for the square 2D chain arrays; the distance between the chains was 40 particles. The particle diameter, array period, and size for the triangular 2D chain arrays were 120 nm, 580 nm, and 300 µm, where the distance of the chains was 33 particles. For the dispersion measurements, the samples were immersed in index-matching oil and sealed with a cover slip. For lasing experiments, a dye solution of IR140 (concentration of 10 mM) or IR792 (concentration of 30 mM) in a 2:1 mixture of benzyl alcohol and dimethyl sulfoxide was prepared. The solvent mixture was chosen such that the refractive index of the dye solution matches that of the substrate. The arrays were immersed in the dye solution by injecting the solution into a cavity of a press-to-seal silicone ring (0.8 mm thickness) pressed between the substrate and a second borosilicate slide.

Lasing and transmission measurement setup

The schematic of the experimental setup used for transmission and lasing experiments is shown in Fig. S1. In transmission measurements, the sample is illuminated with a white light source. The signal from the array is imaged with an NA 0.3 objective, whose back focal plane is imaged onto a spectrometer slit. The back focal plane contains the information about angular distribution of the emission and hence each

position of the slit corresponds to a specific angle θ_y, related to the in-plane wavevector by $k_y = k_0 \sin(\theta_y)$, where $k_0 = 2\pi/\lambda_0$, with the free space wavelength λ_0. The grating inside the spectrometer disperses the light onto a 2D CCD sensor, which then resolves the wavelength along one axis and the wavevector along the other. In addition, real and momentum space images were taken with two CMOS cameras. In the lasing measurements, the sample is optically pumped with ultrafast laser pulses (800 nm central wavelength, 1 kHz repetition rate) through the objective. A long pass filter (cut-off wavelength 850 nm) was used in the detection path to filter out the pump signal. The pump polarization is controlled by a combination of a half-wave plate and a linear polarizer, whereas a neutral density wheel is used to control the pump fluence.

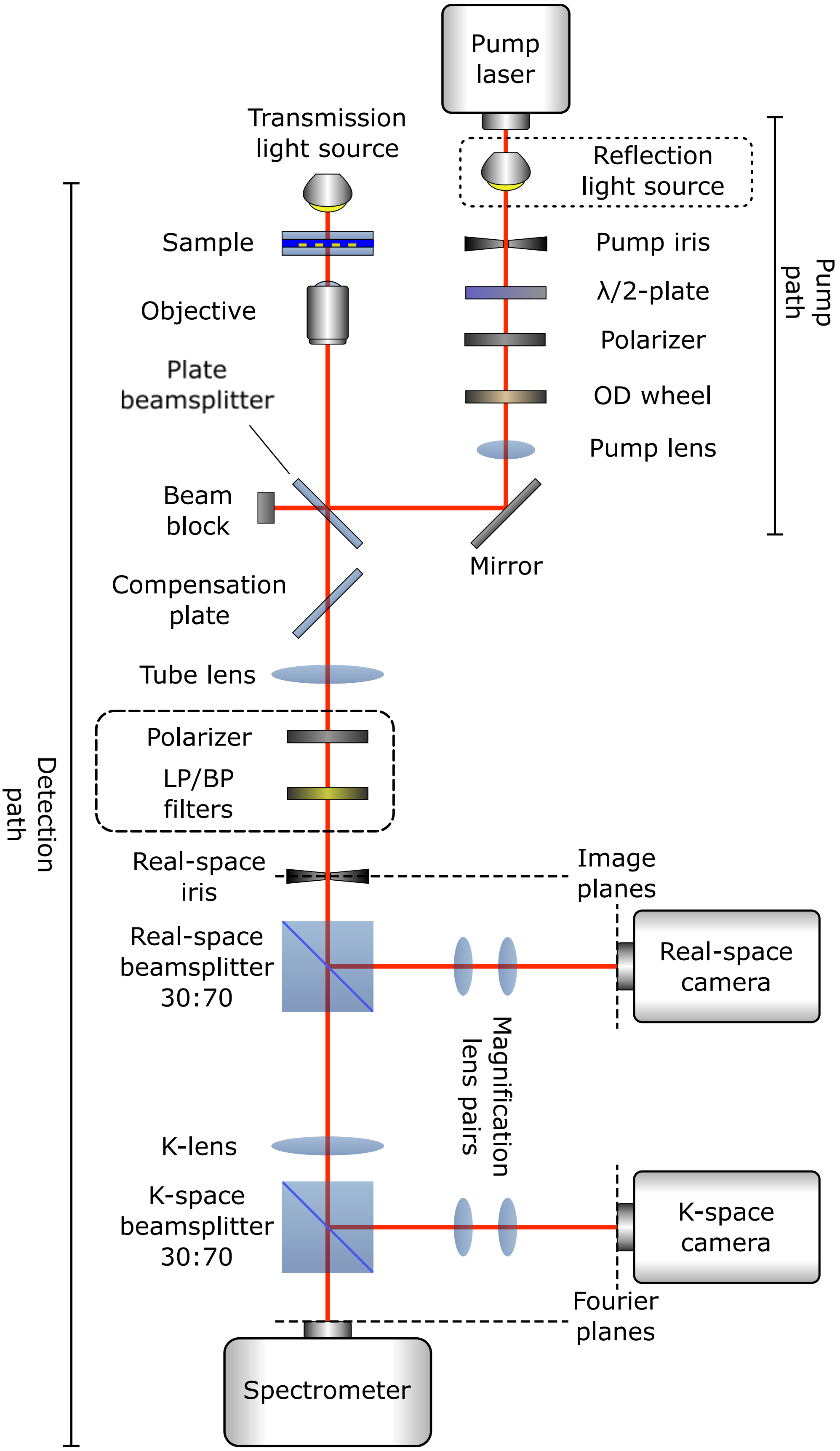

Supplementary Fig. 1 Schematic of the setup used for transmission and emission measurements.

Spatial coherence setup

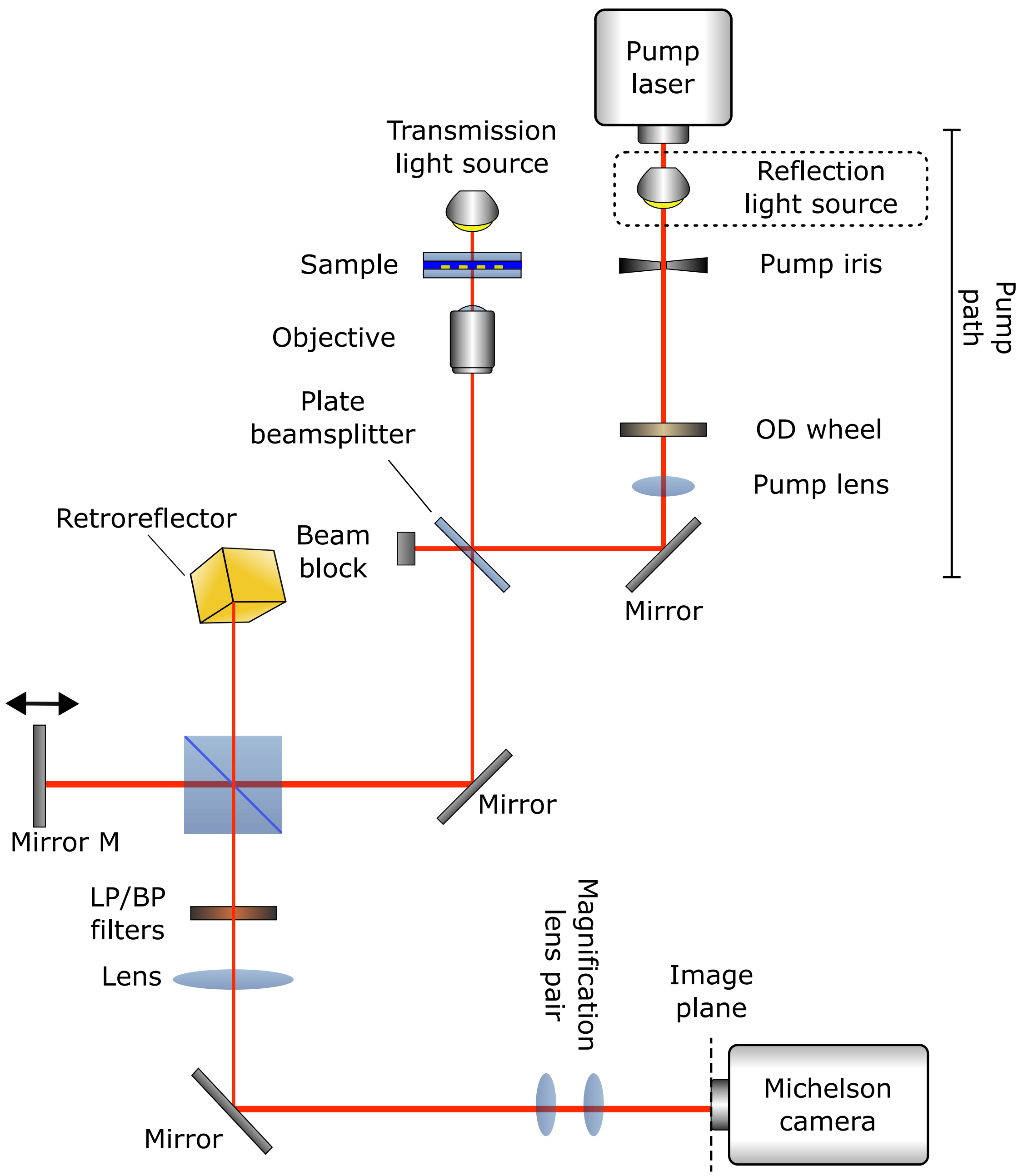

Supplementary Fig. 2 Schematic of the Michelson setup used for spatial coherence measurements. The movable mirror relevant to the interferometry is marked with Mirror M.

Spatial coherence measurement principle

The spatial coherence was measured by routing the emission from the sample with a mirror to a Michelson setup, see Fig. S2.Here, the light is split into two paths, with the first ending on a retroreflector that flips the real-space emission image along the vertical and horizontal axes. Along the second path, the image is guided to a mirror on a movable stage. Both images, i.e., the flipped and unflipped image of the real space emission, are focused onto a CMOS camera, where the interference image is collected. Mirror M is moved until fringes are most visible in the interference image, indicating the point of zero time delay. Since the

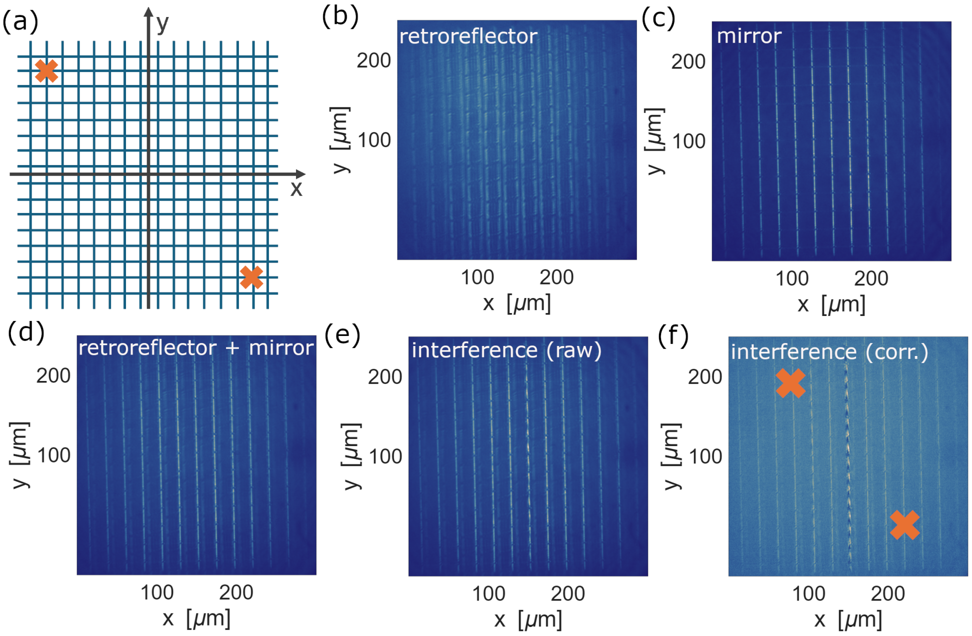

Supplementary Fig. 3 Principle of the Michelson setup used in measurements. (a) The retroreflector flips the image along the vertical and horizontal axes, so that the coherence between pairs of two locations is probed. A pair of such locations is indicated by the two crosses. (b) Retroreflector image (Mirror M path blocked). (c) Mirror M image (retroreflector path blocked). (d) Sum of the intensities of the Mirror M and retroreflector images, i.e., background emission. (e) Raw data of the interference emission (both paths open). (f) Interference background corrected, i.e., (d) is subtracted from (e). The symbols mark the places in this image that correspond to coherence between the two points marked in (a).

retroreflector flips the image of the real space image with respect to the horizontal and vertical axes, we probe coherence of the real space emission between points (x, y) and $(-x, -y)$, as indicated by the crosses in Fig. S3 (a). In total, three measurements are taken: the image of the retroreflector only (blocking the path to Mirror M [Fig. S3 (b)] and the image of the mirror only (blocking the path to the retroreflector) [Fig. S3 (c)]. The sum of these measurements constitutes background emission [Fig. S3 (d)], i.e., no interference effects since one of the beams was blocked in each measurement. The third measurement taken is the interference measurement, where both paths are open Fig. S3 (e). While the fringes are already faintly visible in the raw data of interference, they become clearer after background removal [Fig. S3 (f)]. The visibility of fringes at different locations of this image proves coherence between two specific locations in the original emission, see Figures S3 (a) and (f) for an example.

Theory

The empty lattice approximation consists of imposing the periodicity of the lattice on the free space dispersion of light and of weighting the contribution of each mode with its structure factor [?]. The structure factor $S(\mathbf{k})$ of the mode characterized by the wavevector $\mathbf{k}$ is defined as

$$S(\mathbf{k}) = \frac{1}{N^2} \left| \sum_{n=0}^{N-1} e^{i\mathbf{k}\cdot\mathbf{r}} \right|^2 , \tag{1}$$

where N is the number of lattice sites and $\mathbf{r}_n$ denotes the position of the nth lattice site. The choice of the normalization factor N^{-2} ensures that the structure factor takes values between zero and one. The dispersion of light in a homogeneous, linear, isotropic optical background for in-plane modes is

$$E = \frac{\hbar c_0}{n} \sqrt{k_x^2 + k_y^2}, \tag{2}$$

where $\hbar$ is the reduced Planck constant, c_0 the speed of light in vacuum, and n the refractive index of the background medium. In the (k_x, k_y, E) space, this dispersion describes the surface of a light cone emanating from the point $(k_x, k_y, E = 0)$. Thus, modes that can scatter light to a particular point (k_x, k_y, E) lie on a ring centered at (k_x, k_y) with the radius determined by E. The total scattering intensity is thus proportional to the weighted sum of these modes

$$I(\mathbf{k}_\parallel, E) \propto \int S(\mathbf{k}')\delta(|\mathbf{k}' - \mathbf{k}_\parallel| - \kappa)d\mathbf{k}', \tag{3}$$

where the integration is over the k_x-k_y plane, $\mathbf{k}_\parallel = (k_x, k_y)^{\mathrm{T}}$, and $\kappa = \frac{n}{\hbar c_0}E$ is the wavevector magnitude corresponding to the energy E. Numerically, the above integral is computed by discretizing the k space and summing $S(\mathbf{k})$ over the points for which $||\mathbf{k}' - \mathbf{k}_\parallel| - \kappa| \leq \frac{\Delta E}{2}$, where ΔE is the energy discretization parameter.

If the lattice is taken to be infinite and periodic, the structure factor can take non-zero values only at the sites of the reciprocal lattice $\mathbf{k} = q_1\mathbf{b}_1 + q_2\mathbf{b_2},\ q_1, q_2, \in \mathbb{Z}$, where $\mathbf{b}_{1,2}$ are the reciprocal lattice vectors [?]. The structure factor is then only computed at the sites of the reciprocal lattice. For periodic and infinite lattices, we may forgo computing the integral of Eq. (3) and instead visualize the band structure of the lattice by plotting the dispersion given by Eq. (1) for each mode $(m2\pi/a_x, m'2\pi/a_y)$. In addition, we indicate the polarization of the mode (k_x, k_y) by its TE-polarization fraction [25]

$$p_{\mathrm{TE}} \equiv \begin{cases} 1, \text{ if } k_x = k_y = 0, \\ \frac{|k_y|}{|k_y| + |k_x|}, \text{ otherwise.} \end{cases} \tag{4}$$

This definition assumes the plane of incidence to be the yz plane; if the xz plane is taken to be the plane of incidence, the x and y components are swapped. For dipolar scatterers, such as small plasmonic nanoparticles, diffraction in (out) of the plane of incidence corresponds to linearly TE (TM) polarized light.

All empty lattice calculations have been carried out using an in-house-developed code.

Influence of pump polarization

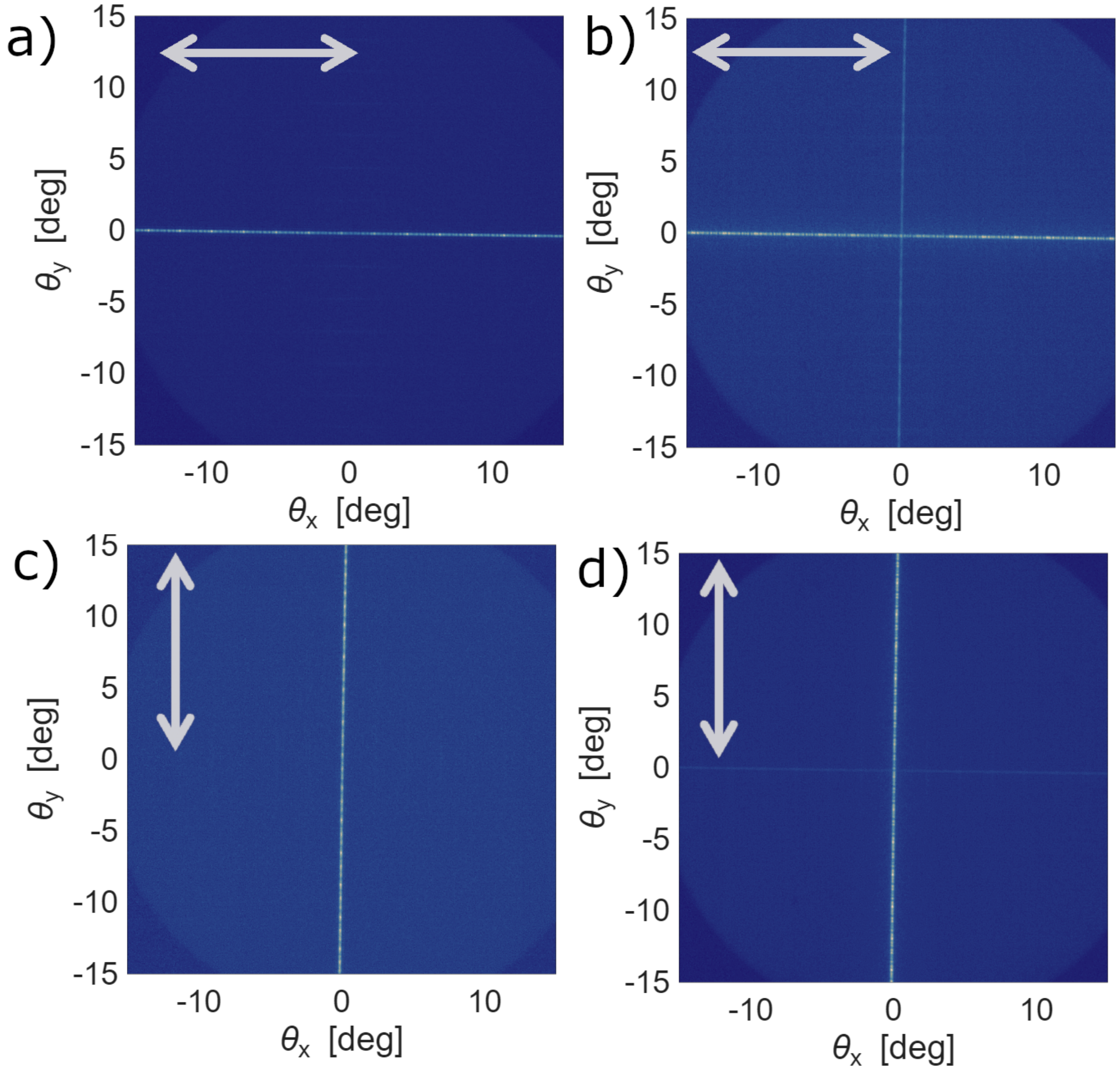

Supplementary Fig. 4 Momentum space emission from square 2D chain arrays with IR140 dye under different pump polarizations. (a)–(b) x-directional pump polarization at fluences of 0.19 and 0.42 mJ/cm^2 (c)–(d) y-directional pump polarization at fluences of 0.20 and 0.45 mJ/cm^2, respectively. The nanoparticles were of a diameter of 110 nm and a height of 50 nm. The array period was 580 nm, and the distance between chains was 40 nanoparticles. Depending on the pump polarization, first, the flat band parallel to the pump polarization is excited, and with higher fluences, the flat band in the other direction appears.

Square 2D chain arrays with a solution of IR140 as a gain medium were pumped with different pump polarizations. Two regimes are visible, where first the emission comes from one flat band, and then from the

second one. Depending on the pump polarization, the flat band oriented parallel to the pump polarization starts emitting first, see Fig. 4.

$\boldsymbol{k}$-space emission data from square chain lattices

Figure 5 shows that the k-space emission from 20x20 (left column) and 30x30 (right column) square chain lattices features a flat band along $k_x = 0$. The notation 20x20 (30x30) indicates the number of particles in the unit cell along the x direction (first number) and y direction (second number). Averaged over k_x, the emission shows periodic features in k_y. The k_y-separation of the maxima corresponds to the length of the reciprocal lattice vector. Curiously, both lattices feature a minimum at the Γ point. We speculate that this is caused by the particular lattice structures, where the lattice sites are distributed symmetrically around but not at the origin [see inset of Fig. 5(a)]. This also results in a "missing" mode at $(k_x, k_y) = \mathbf{b_2} = (0, \frac{2\pi}{Na})$, where N is the number of lattice sites per chain in the lattice and a their spacing, in the band structure of the lattice [panels (c) and (f)]. Interestingly, the emission maxima of the 20x20 square chain lattice are displaced from the Γ point by half of the reciprocal lattice vector's length. These positions correspond to the lowest-energy crossings of the flat band modes. For the 30x30 square chain lattice, the maxima are situated at integer multiples of the reciprocal lattice wavevector; these points correspond to the minima of the flat band modes and their second-lowest energy crossings. Because the k-space data is not spectrally resolved, we cannot further determine to which features of the band structure the maxima correspond. Overall, the features observed in Fig. 5 confirm the origin of the flat band to be consistent with the theoretical description given in [25].

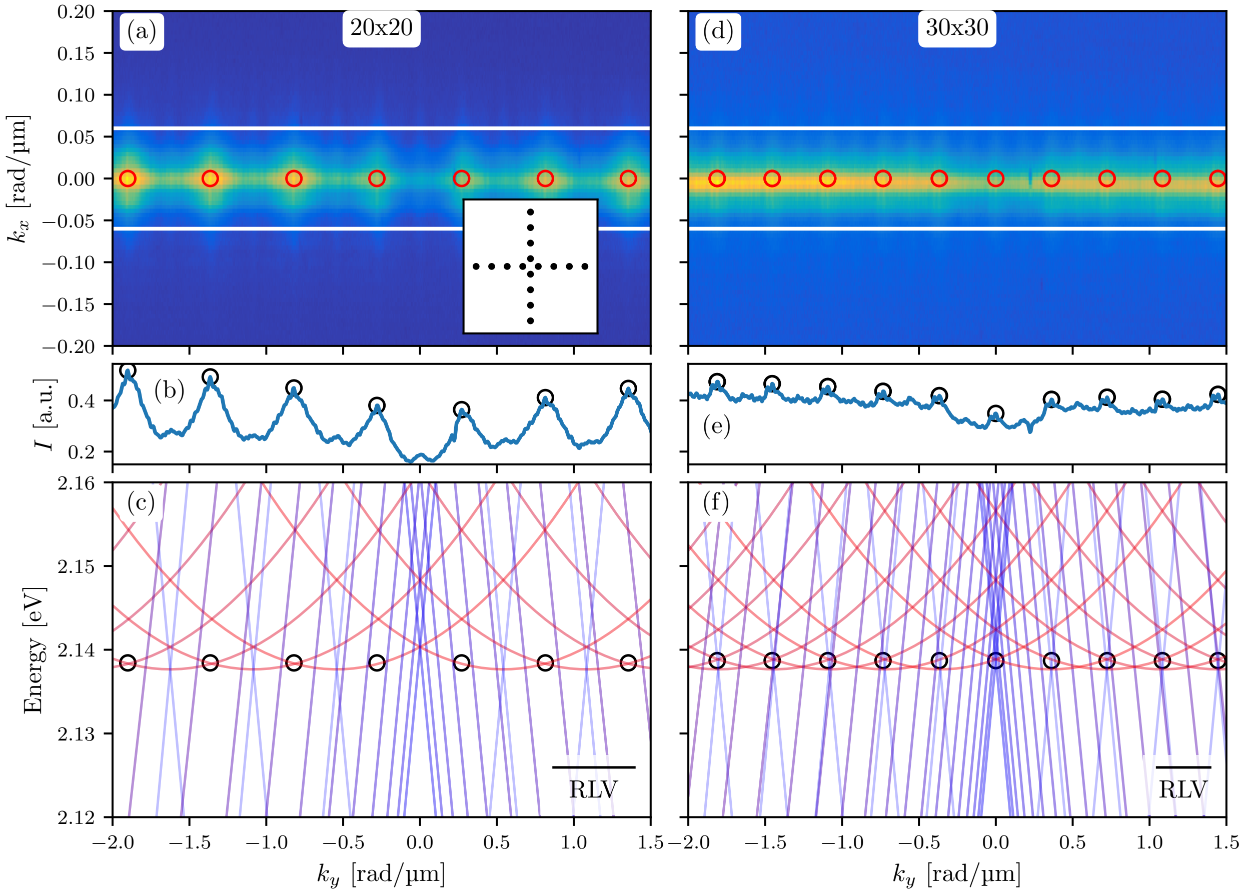

Supplementary Fig. 5 The k-space images of the emission averaged over many shots from a 20x20 square chain lattice (a) and 30x30 square chain lattice (d) show flat bands along the line $k_x = 0$. Blue (yellow) color corresponds to emission minimum (maximum). The inset of panel (a) shows a part of the unit cell of the lattice. Flat bands of both arrays show periodic fluctuations in the emission intensity I averaged over k_x as a function of k_y [panels (b) and (e)] whose period corresponds to the reciprocal lattice vector of the lattice (i.e., the inverse of the unit cell size). The horizontal white lines in panels (a) and (d) indicate the k_x-averaging limits. The k_y positions of the maxima (indicated by circles in all panels) correspond to crossings of the TM modes that form the flat band in the band structure of the lattices calculated using the empty lattice approximation [panels (c) and (f)]. The spectral positions of the circles indicating the maxima in panels (c) and (f) have been set to the energy of the flat band. The lattice site spacing for both lattices was 580 nm. The scale bar RLV in panels (c) and (f) shows the magnitude of the reciprocal lattice vector. In panels (c) and (f), the color of the lines indicates the polarization of the mode calculated using Eq. (4), with blue (red) corresponding to TE (TM) polarization.

Emission from square 2D chain arrays with IR792 dye

The lasing experiments were repeated for the square 2D chain arrays with a different dye molecule (IR 792). Similar to the triangular 2D chain arrays discussed in the main text, we observe two distinct regimes for different pump fluences: for lower pump fluences, emission stems from one flat band mode, for higher pump fluences, emission comes from the other flat band, see Fig. 6.

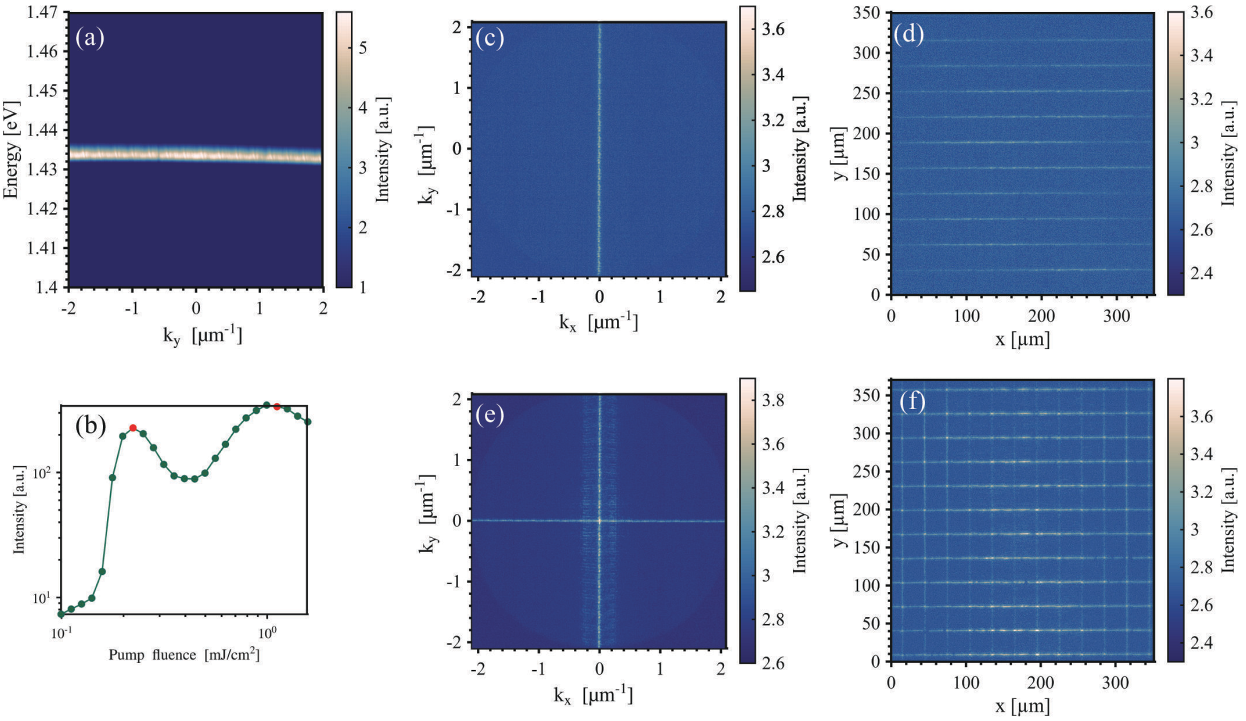

Supplementary Fig. 6 Emission from square 2D chain arrays with IR792 dye. (a) Momentum space resolved spectrum of square 2D chain array emission. (b) Emission dependence on the pump fluence, the red dots denote pump fluence values used for momentum and real space pattern collection. (c)–(f) Real [(c) and (d)] and momentum [(e) and (f)] space emission patterns of square 2D chain arrays for the fluence values of 0.22 mJ/cm^2 [(c) and (e)] and 1.12 mJ/cm^2 [(d) and (f)]. The period of the individual chains and the diameter of the nanoparticles were 564 nm and 120 nm, respectively.
